# Compensating for age limits through emotional crossmodal integration

**DOI:** 10.3389/fpsyg.2015.00691

**Published:** 2015-05-27

**Authors:** Laurence Chaby, Viviane Luherne-du Boullay, Mohamed Chetouani, Monique Plaza

**Affiliations:** ^1^Institut de Psychologie, Sorbonne Paris Cité, Université Paris Descartes, Boulogne-Billancourt, France; ^2^Groupe Intégration Multimodale, Interaction et Signal Social, Institut des Systèmes Intelligents et de Robotique, CNRS UMR 7222, Paris, France; ^3^Université Paris 8 Vincennes Saint Denis, Saint-Denis, France

**Keywords:** aging, emotion, faces, voices, non-verbal vocalizations, multimodal integration, race model

## Abstract

Social interactions in daily life necessitate the integration of social signals from different sensory modalities. In the aging literature, it is well established that the recognition of emotion in facial expressions declines with advancing age, and this also occurs with vocal expressions. By contrast, crossmodal integration processing in healthy aging individuals is less documented. Here, we investigated the age-related effects on emotion recognition when faces and voices were presented alone or simultaneously, allowing for crossmodal integration. In this study, 31 young adults (*M* = 25.8 years) and 31 older adults (*M* = 67.2 years) were instructed to identify several basic emotions (happiness, sadness, anger, fear, disgust) and a neutral expression, which were displayed as visual (facial expressions), auditory (non-verbal affective vocalizations) or crossmodal (simultaneous, congruent facial and vocal affective expressions) stimuli. The results showed that older adults performed slower and worse than younger adults at recognizing negative emotions from isolated faces and voices. In the crossmodal condition, although slower, older adults were as accurate as younger except for anger. Importantly, additional analyses using the “race model” demonstrate that older adults benefited to the same extent as younger adults from the combination of facial and vocal emotional stimuli. These results help explain some conflicting results in the literature and may clarify emotional abilities related to daily life that are partially spared among older adults.

## Introduction

Emotion recognition is a fundamental component of social cognition. The ability to discriminate and interpret others’ emotional states from emotional cues plays a crucial role in social functioning and behaviors (Carton et al., 1999; Adolphs, 2006; Corden et al., 2006; Frith and Frith, 2012). From early and throughout lifespan, emotion recognition is an essential mediator of successful social interactions and well-being (Izard, 2001; Engelberg and Sjöberg, 2004; Kryla-Lighthall and Mather, 2009; Suri and Gross, 2012). Hence, impaired recognition of others’ emotional states may result in severe social dysfunctions, including inappropriate social behaviors, poor interpersonal communication and reduced quality of life (Feldman et al., 1991; Shimokawa et al., 2001; Blair, 2005). Such difficulties have been observed not only in disorders characterized by prominent social-behavioral deficits (i.e., autism spectrum disorders, schizophrenia, neurodegenerative dementia; e.g., Chaby et al., 2012; and see for review Kennedy and Adolphs, 2012; Kumfor et al., 2014) but also in normal aging, which is frequently associated with social withdrawal and loneliness (e.g., Szanto et al., 2012; Steptoe et al., 2013).

Although older adults report high levels of satisfaction and better emotional stability with advancing age (Reed and Carstensen, 2012; Sims et al., 2015), they have difficulties processing some types of emotional information, which is often marked by a decline in emotion recognition (Ruffman et al., 2008; Isaacowitz and Blanchard-Fields, 2012). Most past studies have identified age-related difficulties in the visual channel, particularly when participants were asked to recognize emotion from posed facial expressions (see for review, Chaby and Narme, 2009; Isaacowitz and Stanley, 2011). These posed expressions were created to convey a single specific emotion, typically with exaggerated individual features, without any distracting or irrelevant features. However, emotions are not usually expressed solely by the face during daily social interactions; typically, voice (including non-verbal vocalizations) is also an important social signal, which needs to be processed quickly and accurately to allow successful interpersonal interactions. The rare studies that have explored how the ability to recognize vocal emotion changes with age have been conducted on speech prosody using words or sentences spoken with various emotional expressions. Theses studies concluded that advancing age is associated with increasing difficulties in recognizing emotion from prosodic cues (Kiss and Ennis, 2001; Paulmann et al., 2008; Mitchell et al., 2011; Lambrecht et al., 2012; Templier et al., 2015). However, vocal emotions could also be experienced via non-verbal affect bursts (e.g., *screams or laughter*; see Scherer, 1994) that typically accompany intense emotional feelings and that might be considered as the vocal counterpart of facial expressions. The processing of non-verbal vocal affects in aging individuals has rarely been studied (see Hunter et al., 2010; Lima et al., 2014); thus, this issue needs to be further investigated.

Altogether, the above studies showed evidence of age-related decline of some basic emotions via unimodal visual or auditory channels. These changes might start early, at approximately 40 years, for both facial (Williams et al., 2009) and prosodic emotions (Paulmann et al., 2008; Mill et al., 2009; Lima and Castro, 2011), and decline may occur linearly with advancing age (see Isaacowitz et al., 2007). In particular, compared to young adults, older adults could experience difficulties recognizing fear, anger and sadness from faces but experience no deficits recognizing happy or neutral faces (see for review, Isaacowitz et al., 2007; Ruffman et al., 2008). The recognition of disgust also seems highly preserved in older adults (e.g., Calder et al., 2003). Data from voices are less coherent, as difficulties have been found in older adults only for anger and sadness (Ruffman et al., 2008) or for almost all emotions (e.g., Paulmann et al., 2008).

Different mechanisms have been proposed to explain these age-related changes in emotion recognition. One preeminent explanation concerns structural and functional brain changes associated with age. Multiple interconnected brain regions are implicated in visual and auditory emotional processing. These regions include the frontal lobes, particularly the orbitofrontal cortex (Hornak et al., 2003; Wildgruber et al., 2005; Tsuchida and Fellows, 2012) and the temporal lobes, particularly the superior temporal gyrus (Beer et al., 2006; Ethofer et al., 2006). The amygdala is also involved in this processing (Iidaka et al., 2002; Fecteau et al., 2007). Prefrontal cortex atrophy (in particular atrophy of the orbitofrontal region; Resnick et al., 2003, 2007; Lamar and Resnick, 2004) is a known marker of normal aging and could explain the difficulties identifying some facial emotions, in particular anger. Moreover, although the amygdala does not decline as rapidly as the frontal regions, some studies have reported a linear reduction of its volume with age (Mu et al., 1999; Allen et al., 2005). When comparing elderly people with young adults, neuroimaging studies observed a less significant activation of this structure among the elderly during the processing of emotional faces, especially negative ones (Mather et al., 2004). This was coupled with increased activity in the prefrontal cortex (Gunning-Dixon et al., 2003; Urry et al., 2006; Ebner et al., 2012). Conversely, other studies found a decrease in functional connectivity between the amygdala and posterior structures, which may reflect a decline in the perceptual process (Jacques et al., 2009). Overall, these patterns of brain activity observed in neuroimaging studies during a variety of emotional tasks (including recognition) are consistent with the Posterior–Anterior Shift in Aging (PASA; for review, see Dennis and Cabeza, 2008), which reflects the effect of aging on brain activity.

Another explanation for older adults’ lower performance on negative emotion recognition emerges within the framework of the socio-emotional selectivity theory (Carstensen, 1992). With advancing age, adults appear to concentrate on a few emotionally rewarding relationships with their closest partners, report greater emotional control, and reduce their cognitive focus on negative information. Based on these observations, it was suggested that “paradoxically,” the recognition of negative emotion declines (Carstensen et al., 2003; Charles and Carstensen, 2010; Mather, 2012; Huxhold et al., 2013).

Losses in cognitive and sensory functions are also possible explanations for age-related changes in emotion recognition. Increasing age is often associated with a decline in cognitive abilities (e.g., Verhaeghen and Salthouse, 1997; for review, see Salthouse, 2009), as well as with losses in visual and auditory acuity (Caban et al., 2005; Humes et al., 2009), which could hamper higher-level processes such as language and perception (Sullivan and Ruffman, 2004). However, these sensory attributes are shown to be poor predictors of the age-related decline in visual or auditory emotional recognition (e.g., Orbelo et al., 2005; Mitchell, 2007; Ryan et al., 2010; Lima et al., 2014).

Previous research on age-related differences in the recognition of basic emotions has focused predominantly on a single modality, and thus little is known about age-related differences in crossmodal emotion recognition. However, in daily life, people perceive emotions through multiple modalities, such as speech, voices, faces and postures (e.g., Young and Bruce, 2011; Belin et al., 2013). This indicates that our brain merges information from different senses to enhance perception and guide our behavior (Ernst and Bülthoff, 2004; Ethofer et al., 2013). Evidence supporting this idea includes studies of brain-damaged patients, such as traumatic or vascular brain injuries and brain tumors. These studies found similar impairments in processing emotions from faces and voices in a single modality, but found that brain-damaged patients experienced greater performance using both facial and vocal stimuli (e.g., Hornak et al., 1996; Borod et al., 1998; Calder et al., 2001; Kucharska-Pietura et al., 2003; du Boullay et al., 2013; Luherne-du Boullay et al., 2014).

Some studies in young adults have demonstrated that congruent emotional information processed via multisensory channels optimizes behavioral responses, which results in enhanced accuracy and faster response times (RT; De Gelder and Vroomen, 2000; Kreifelts et al., 2007; Klasen et al., 2011). In older adults, audio-visual performances have been shown to be equivalent or even improved relative to younger adults (Laurienti et al., 2006; Peiffer et al., 2007; Diederich et al., 2008; Hugenschmidt et al., 2009; DeLoss et al., 2013), with more rare exceptions showing reduced multisensory integration in older adults (Walden et al., 1993; Sommers et al., 2005; Stephen et al., 2010). Some of these studies have explored the effects of age on crossmodal emotional processing and found evidence for preserved multisensory processing in older adults when congruent auditory and visual emotional information were presented simultaneously (Hunter et al., 2010; Lambrecht et al., 2012).

Multisensory integration refers to the process by which unisensory inputs are combined to form a new integrated product (Stein et al., 2010). This process has been studied in humans using neuroimaging techniques, which show that different regions of the human brain are implicated in the integration of multimodal cues, including “convergence” areas such as the superior temporal sulcus (STS; Laurienti et al., 2005; James and Stevenson, 2011; Watson et al., 2014; see for review, Stein et al., 2014). Neuroimaging techniques such as functional magnetic resonance imaging (fMRI) generally show greater activity in response to bimodal stimulation. More precisely, in a series of fMRI experiments conducted by Kreifelts and collaborators (e.g., Kreifelts et al., 2007; see for review, Brück et al., 2011) the posterior superior temporal cortex (p-STC) emerges as a crucial structure for the integration of facial and vocal cues. In event-related potential (ERP) studies (e.g., Giard and Peronnet, 1999; Foxe et al., 2000; Molholm et al., 2002), multisensory enhancement is measured by comparing the ERP from the multisensory condition to the sum of the ERPs from each unimodal condition. Multisensory enhancement is also commonly measured in behavioral studies by calculating a redundancy gain between the crossmodal stimulus and the more informational unimodal stimulus. Another interesting method performed in studies using RT, is to test whether the redundant target effect (shorter RT under the crossmodal condition) reflects an actual multisensory integrative process by comparing the observed RT distribution with the distribution predicted by the “race model” (proposed by Miller, 1982; see also Colonius and Diederich, 2006). The “race model” assumes that a crossmodal stimulus presentation produces parallel activation (i.e., in a separate way) of the unimodal stimuli. According to this model, the shortening of RT for crossmodal relative to unimodal stimuli derives from the fact that either unimodal stimulus can produce a response. Thus, any violation of the race model (i.e., if the observed RTs in crossmodal trials are shorter than those predicted by the race model) indicates that the stimuli are not processed in separate channels, which suggests an underlying integrative mechanism (see Laurienti et al., 2006; Girard et al., 2013; Charbonneau et al., 2013).

To date, the processing mechanisms responsible for multisensory enhancement in older compared to young adults remains unclear, and crossmodal emotional integration in aging evaluated by the race model has not been investigated. To characterize the age-related effect on emotional processing, we used emotional human stimuli (i.e., happy, angry, fear, sad and disgust) and a neutral expression in the form of unimodal (facial or vocal) or crossmodal (simultaneous congruent facial and vocal expressions) cues. Isolated facial expression was studied using pictures of posed facial expressions, and isolated vocal expression was studied using non-verbal affect stimuli. Our primary focus concerned crossmodal emotional processing in aging, and we aimed to explore whether older adults benefit from congruent crossmodal integration and to better understand the nature of this benefit. According to recent studies of multisensory integration mechanisms during aging (e.g., Lambrecht et al., 2012; Freiherr et al., 2013; Mishra and Gazzaley, 2013), we hypothesized that older adults benefit from congruent crossmodal presentation when identifying emotions. To assess this hypothesis, we calculated redundancy gains for scores and used the race model for RTs to determine the nature of multisensory integration achieved by combining redundant visuo-auditory information.

## Materials and Methods

### Participants

The study participants consisted of 31 younger (20–35; *M* = 25.8, SD = 6.4; 16 females) and 31 older adults (60–76; *M* = 67.2, SD = 5.8; 17 females); see Table 1. The participants spoke French and reported having normal or corrected-to-normal vision and good hearing abilities at the time of testing. All participants were living independently in the community and were in good general physical health. None of the participants had any history of psychiatric or neurological disorders, which might compromise cognitive function. They also had a normal score on the Beck Depression Inventory (Beck et al., 1996; BDI II, 21 item version; a score of less than 17 was considered to be in the minimal range). All elderly adults completed the Mini Mental State Examination (Folstein et al., 1975; MMSE), on which they scored above the cut-off score (26/30) for risk of dementia. Grade level was calculated with the Mill Hill Vocabulary Scale (French adaptation: Deltour, 1993), and this did not differ between groups (*p* = 0.55).

**TABLE 1:** **Participant demographic characteristics**.

****	**Younger adults (*n* = 31)**	**Older adults (*n* = 31)**
Age (years)	25.8 ± 6.4	67.2 ± 5.8
Education (years)	14.18 ± 1.6	13.55 ± 2.8
Mill-hill	36.87 ± 3.0	37.48 ± 4.8
Sex ratio (M/F)	16/15	17/14
BDI-II (/63)	5.65 ± 6.5	5.25 ± 3.8
MMSE (/30)	–	29.33 ± 0.6

The study was approved by the ethics committee of Paris Descartes University (Conseil d’Evaluation Ethique pour les Recherches en Santé, CERES, n IRB 2015100001072) and all participants gave informed consent.

### Materials

**FIGURE 1:**
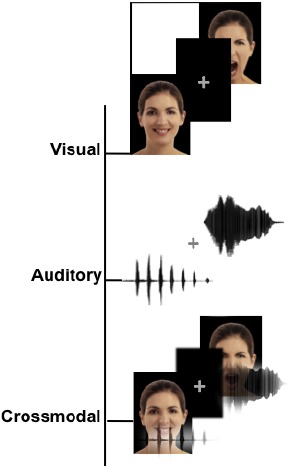
**Schematic representation of the stimuli.** Examples of the stimuli for the three different modalities, including visual (facial expressions), auditory (non-verbal affective vocalizations) and crossmodal stimuli (congruent facial and vocal emotions presented simultaneously).

Examples of stimuli and the task design for each condition are illustrated in Figure 1.

*Visual stimuli*. Visual stimuli consisted of pictures of human facial expressions obtained from the Karolinska Directed Emotional Faces database (Lundqvist et al., 1998). This database was chosen because it provided good examples of universal emotion categories with a high accuracy of labeling. The faces of 10 models (5 females, 5 males) expressing facial expressions of happiness, sadness, anger, fear, disgust or neutral constituted a set of 60 stimuli. All stimuli (presented on a black background) were 10 cm in height and subtended a vertical visual angle of 8° at a viewing distance of 70 cm.

*Auditory stimuli*. Auditory stimuli (Figure 1) consisted of non-verbal affective vocalizations (cry, laugh, etc.) obtained from The Montreal Affective Voices database (Belin et al., 2008). This database was chosen because it provided a standardized set of emotional vocalizations corresponding to the universal emotion categories without the potential confounds from linguistic content. The voices of 10 actors (5 females, 5 males) expressing happiness, sadness, anger, fear, disgust or neutral, vocalization constituted a set of 60 stimuli.

*Crossmodal stimuli*. Each emotional face was combined with an affective vocalization to construct 60 congruent expressions of faces and voices. The gender of the face and the voice were always congruent.

### Procedure

Participants were tested individually in a single session that lasted approximately 45 min. The protocol was run using E-prime presentation software (Psychology Software Tools). Prior to the experiment, short facial-matching and vocal-matching tasks were administered to control for basic visual and auditory abilities in processing faces and voices. The subjects were asked to match the identity of non-emotional faces (i.e., six pairs of neutral faces obtained from the Karolinska Directed Emotional Faces Database) and non-emotional voices (i.e., six pairs of neutral voices obtained from the Montreal Affective Voices database). The stimuli were different from those used in the main task.

Then, after a short familiarization period, the experiment began. The experiment consisted of three blocks (visual, auditory, crossmodal) of 60 trials. Each trial started with the presentation of a fixation cross for 300 ms and was followed by the target stimulus, which was presented or repeated until the subject responded. Participants were asked to select (by clicking with the computer mouse) one label from a list of choices that best described the emotion presented. The six labels were displayed at the bottom of the computer screen and were visible throughout the test. There was an inter-trial interval of 700 ms. The order of the three blocks was counterbalanced across participants, and the order of trials was pseudo-randomized across each block. During the session, resting pauses were provided after every 10 trials, and the participants could take breaks if necessary between blocks. No feedback was given to the participants.

### Statistical Analysis

Participants’ accuracy (scores of correct responses) and corresponding RT (in milliseconds, ms) was computed for each condition. To control for outliers, trials with RT below 200 ms or greater than two standard deviations above the mean of each condition (0.90% of the trials in young adults; 1.25% of the trials in older adults) were excluded.

First, the data were entered into an overall analysis of variance (ANOVA), with age (young adults, older adults) as a between-subjects factor and with modality (visual, auditory, crossmodal) and emotion (neutral, happiness, fear, anger, sadness, disgust) as within-subjects factors. Effect sizes are reported as partial eta-squared (${\text{η}}_{p}^{2}$). ANOVAs were adjusted with the Greenhouse-Geisser non-sphericity correction for effects with more than one degree of freedom. To provide clarity, uncorrected degrees of freedom, the Greenhouse-Geisser epsilon (ε) and adjusted *p* values are reported. Planned comparisons or *post hoc* Bonferroni tests were conducted to further explore the interactions between age, modality and emotion. The alpha level was set to 0.05 (*p* values were corrected for multiple comparisons).

Second, to examine whether both groups showed redundancy gains, as reflected by the difference in the scores when the visual and auditory stimuli were presented together (crossmodal condition) compared to each modality alone (unimodal condition), we calculated a “redundancy gain” for each participant separately by subtracting the higher of the scores under the unimodal conditions from the score under the crossmodal condition [(*crossmodal score—best modality score) × 100*] (see Calvert et al., 2004; Girard et al., 2013). The significance of the difference in redundancy gain (*in percent*) between younger and older participants was tested using an independent samples *t* - test.

Finally, to further test the advantage of crossmodal over unimodal processing, we investigated whether the RTs obtained under the crossmodal condition exceeded the statistical facilitation predicted by the race model (Miller, 1982). In multisensory research, the race model inequality has become a standard tool to identify crossmodal integration using RT data (Townsend and Honey, 2007). To analyze the race model inequality, we used RMItest software (http://psy.otago.ac.nz/miller), which implements the algorithm described in Ulrich et al. (2007). The procedure requires four steps. First, participants’ RTs in each condition (i.e., visual, auditory and crossmodal) are converted to cumulative distribution functions (CDFs). Second, the race model distribution is calculated by summing the CDFs of observed responses to the two unimodal conditions (visual and auditory) to create a “predicted” multisensory distribution. Third, percentile points (i.e., in the present study: 5th, 15th, 25th, 35th, 45th, 55th, 65th, 75th, 85th, and 95th) are determined for every distribution of RT. Finally, in each group, the mean RT for the crossmodal condition and the “predicted” condition are compared for each percentile using a *t*-test. If significant values are obtained in the crossmodal condition relative to the predicted condition, we conclude that the race model cannot account for the facilitation of the redundant signal conditions, supporting the existence of an integrative process.

## Results

### Age-related Difference in Emotion Recognition

**FIGURE 2:**
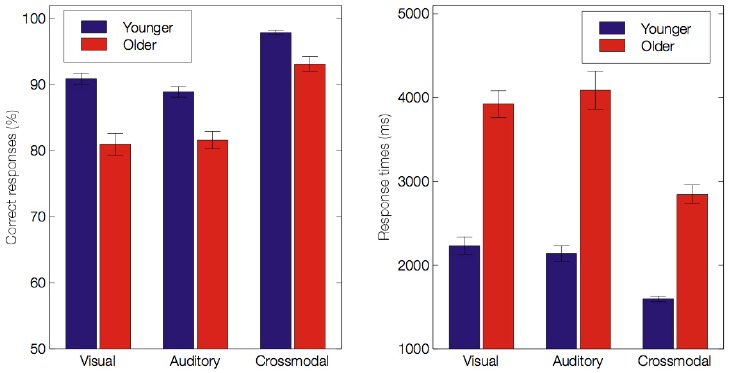
**Mean accuracy scores (%) and response times (ms) for both age groups under the visual, auditory and crossmodal conditions.** Error bars indicate standard errors of the means.

Mean performance and RTs for all conditions are presented in Table 2^1^. For both younger and older groups, the mean performance accuracy was greater than 80% for the visual, auditory and crossmodal conditions. However, we found significant main effects of age, indicating that older adults performed less accurately and more slowly than younger adults (85.23 ± 1.24% vs. 92.58 ± 0.51%, *F*(1,60) = 30.17, *p* < 0.001, ${\text{η}}_{p}^{2}$ = 0.33 for scores; 3619 ± 145 ms vs. 1991 ± 68 ms, *F*(1,60) = 103.4, *p* < 0.001, ${\text{η}}_{p}^{2}$ = 0.63 for RTs). Importantly, we found a significant effect of modality on the scores [*F*(2,120) = 137.54, *p* < 0.001, ε = 0.92, ${\text{η}}_{p}^{2}$ = 0.7] and the RTs [*F*(2,120) = 62.48, *p* < 0.001, ε = 0.88, ${\text{η}}_{p}^{2}$ = 0.51], indicating that participants responded more effectively under the crossmodal condition than under either unimodal condition (all *p* < 0.001). There was a significant effect of emotion on the scores [*F*(5,300) = 92.11, *p* < 0.001, ε = 0.62, ${\text{η}}_{p}^{2}$ = 0.60] and the RTs [*F*(5,300) = 36.91, *p* < 0.001, ${\text{η}}_{p}^{2}$ = 0.38]. Furthermore, main effects were accompanied by several two-way interactions: between group and modality (see Figure 2) on the scores [*F*(2,120) = 6.98, *p* = 0.002, ε = 0.92, ${\text{η}}_{p}^{2}$ = 0.10] and the RTs [*F*(2,120) = 7.66, *p* = 0.001, ε = 0.88, ${\text{η}}_{p}^{2}$ = 0.11]; between group and emotion on the scores [*F*(5,300) = 8.13, ε = 0.61, *p* < 0.001, ${\text{η}}_{p}^{2}$ = 0.12] and the RTs [*F*(5,300) = 8.62, *p* < 0.001, ${\text{η}}_{p}^{2}$ = 0.12], and between modality and emotion on the scores [*F*(10,600) = 27.01, *p* < 0.001, ε = 0.55, ${\text{η}}_{p}^{2}$ = 0.31] and the RTs [*F*(10,600) = 10.52, *p* < 0.001, ε = 0.56, ${\text{η}}_{p}^{2}$ = 0.15]. Importantly, there was a significant effect of the three-way interaction between group, modality and emotion on the scores [*F*(10,600) = 3.23, *p* = 0.005, ε = 0.55, ${\text{η}}_{p}^{2}$ = 0.05] and the RTs [*F*(10,600) = 2.7, *p* = 0.016, ${\text{η}}_{p}^{2}$ = 0.04]. This reveals the following (see Table 2): (a) in the visual and auditory modality, older adults have lower scores than younger adults for the negative emotions only^2^ (i.e., sadness, anger and disgust in the visual modality, *p* < 0.01; anger and fear in the auditory modality, *p* < 0.01), and (b) in the crossmodal condition, older adults perform more poorly than younger adults for anger only (*p* < 0.001). Concerning RTs, in the unimodal and crossmodal conditions, older adults were slower to identify all emotions (all *p* < 0.001) except for happiness (*p* > 0.1).

**TABLE 2:** **Mean accuracy scores (%) and response times (ms) by age group and emotion. Standard errors of the means are shown in parentheses**.

		Mean accuracy (%)					
		**Neutral**	**Happy**	**Fear**	**Sadness**	**Anger**	**Disgust**
**Older**	Visual	90.9 (2.7)	99.3 (0.4)	83.2 (2.3)	73.5 (3.4)	66.1 (4.7)	72.5 (2.5)
	Auditory	92.6 (2.7)	95.2 (1.9)	73.2 (3.9)	94.5 (1.3)	47.7 (3.4)	86.4 (2.3)
	Crossmodal	98.7 (0.6)	100 (0.0)	89.6 (2.1)	95.8 (1.8)	76.8 (4.2)	97.7 (0.8)
**Younger**	Visual	97.1 (1.1)	99.7 (0.6)	87.7 (2.2)	90.9 (2.6)	84.5 (2.4)	85.4 (2.5)
	Auditory	96.7 (0.9)	98.0 (0.9)	86.7 (2.3)	94.2 (1.3)	62.9 (3.0)	94.8 (1.2)
	Crossmodal	99.6 (0.3)	99.0 (0.7)	97.4 (0.9)	97.4 (0.8)	94.5 (1.5)	99.3 (0.4)
		**Response times (ms)**					
		**Neutral**	**Happiness**	**Fear**	**Sadness**	**Anger**	**Disgust**
**Older**	Visual	3447 (227)	2502 (136)	4366 (225)	4805 (325)	4243 (280)	4173 (224)
	Auditory	4355 (342)	3195 (213)	4370 (265)	3824 (238)	4920 (354)	3871 (250)
	Crossmodal	2796 (158)	2188 (105)	3135 (138)	3115 (146)	3273 (190)	2567 (132)
**Younger**	Visual	1871 (107)	1555 (50)	2408 (162)	2464 (157)	2569 (177)	2538 (163)
	Auditory	2148 (157)	1967 (117)	2345 (153)	2082 (74)	2281 (124)	2026 (80)
	Crossmodal	1613 (49)	1370 (35)	1689 (54)	1722 (50)	1571 (45)	1624 (35)

### Integration of Crossmodal Emotional Information in Aging

To explore the ultimate crossmodal gain in the scores, we calculated a “redundancy gain” (i.e., the difference between the crossmodal condition and the unimodal condition with the higher score) for each participant in the two groups (see Materials and Methods section).

For the scores, our analysis indicated that the redundancy gain was greater for the older (8.82%) than for the younger adults (5.86%, *p* = 0.007). In the older group, all but two subjects showed a redundancy gain (29/31; one performed equally between the auditory modality and the crossmodal condition, and the other performed slightly better under the visual condition compared to the crossmodal condition). Moreover, there was a significant difference between the unimodal and crossmodal conditions for all emotions (all *p* < 0.003). In the younger group, all subjects except for one (30/31; who performed equally between the auditory condition and the crossmodal condition) showed a redundancy gain. Our analysis showed a significant difference between the unimodal and crossmodal conditions for negative emotions only (fear, sadness, anger and disgust) (all *p* < 0.007); for the neutral emotion and for happiness, performance ceilings may explain the lack of significant effects (all *p* > 0.1).

**FIGURE 3:**
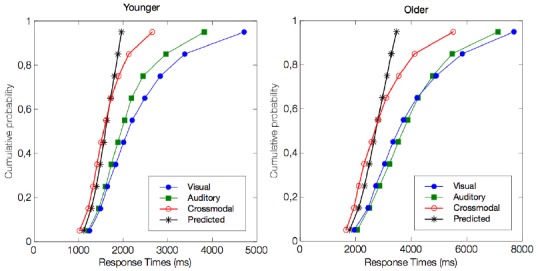
**Test for the violation of race model inequality.** The figure illustrates the cumulative probability curves of the RT under the visual (blue circles), auditory (green squares), and crossmodal conditions (red circles). The summed probability for the visual and auditory responses is depicted by the race model curve (marked by an asterisk). Note that the crossmodal responses are faster than the race model prediction for the four fastest percentiles, i.e., the 5th, 15th, 25th, and 35th percentiles (all *p* < 0.01).

For RTs, we used the race model to explore crossmodal integration and to determine whether the observed crossmodal behavioral enhancement (i.e., shorter RTs) was beyond that predicted by statistical summation of the unimodal visual and auditory conditions (Figure 3). In the younger group, we observed a violation of the race model prediction for the 5th, 15th, 25th, and 35th percentiles of the RT distribution (all *p* < 0.01, but not for the slowest percentiles (all *p* > 0.1). These results support the existence of a crossmodal integrative process. The temporal window in which this benefit was significant was from 1019 to 1410 ms. Similar to the responses by the younger group, the older group responses were shorter than those predicted by the race model for the 5th, 15th, 25th, and 35th percentiles of the RT distribution (all *p* < 0.01). The temporal window in which this benefit was significant was from 1647 to 2300 ms. Although the maximal enhancement occurred at different absolute RTs between the two populations, this peak enhancement occurred at the exact same percentile of the cumulative distribution curve.

## Discussion

While a large body of evidence shows that older adults are less accurate than younger adults in recognizing specific emotions from emotional faces, fewer studies have examined vocal emotion recognition, and hardly any studies have investigated the recognition of emotion from emotional faces and voices presented simultaneously (Hunter et al., 2010; Lambrecht et al., 2012). The purpose of this study was to compare unimodal facial and vocal emotion processing in older and younger adults and, in addition, to test whether older adults benefit from the combination of congruent emotional information from different channels, which reveals crossmodal integration. Our results first confirm that older adults experience difficulties in emotion recognition. They were less accurate and slower overall than younger adults in processing emotion from facial or non-verbal vocal expressions presented alone. Second, the participants similarly recognized facial and vocal cues, and both groups benefitted from the crossmodal condition. Third, age-related differences were modulated by emotion, as older adults were particularly affected in term of accuracy with regards to processing negative emotions under both the facial and vocal conditions. Finally, our results provide compelling evidence for the multisensory nature of emotional processing in aging. The important finding of this study was that older adults benefit to the same extent as younger adults from the combination of information presented in the visual and auditory modalities. This suggests that crossmodal processing represents a mechanism compensating for deficits in the visual or auditory channels that often affect older adults.

### Effects of Age on Emotion Recognition Based on Unimodal Stimuli

Our findings indicated that emotion recognition based on unimodal stimuli changes with age. In the visual modality, our results support previous findings showing age-related difficulties in the ability to recognize emotion from facial cues (see for a meta-analysis, Ruffman et al., 2008). However, most of these studies used the collection of posed black-and-white photographs of human faces from the 1970s Ekman dataset (e.g., Orgeta and Phillips, 2008; Hunter et al., 2010; Slessor et al., 2010) that has been criticized for its lack of ecological validity, which leads to questions about the generalizability of the results (Murphy and Isaacowitz, 2010). The present study used emotional expressions consisting of static color photographs of faces (see also, Ebner et al., 2010; Eisenbarth and Alpers, 2011), and this study confirmed the robustness of age-related difficulties. The fact that the same results were found using dynamic facial expressions (Lambrecht et al., 2012) confirms that widespread difficulties in recognizing emotion from facial cues are encountered by older adults. In the auditory modality, the ability to recognize emotion from non-verbal vocal cues also becomes less efficient with age. This result is in accordance with that of Hunter et al. (2010), who used non-verbal affective vocalizations. It is also in line with some recent studies using spoken words in a neutral context that showed impairments in decoding emotional speech with advancing age (Paulmann et al., 2008; Mitchell et al., 2011; Lambrecht et al., 2012). As normal variations of prosodic emotion ability could be associated with depression, relationship satisfaction or well-being in younger populations (Noller and Feeney, 1994; Emerson et al., 1999; Carton et al., 1999), the question remains whether and how the age-related decline in emotional vocal processing influences social interactions. However, it is important to note that in our study, the performance of the older group reached 80%, suggesting a relatively mild deficit. This suggest that non-verbal vocalizations, that are devoid of linguistic information, are however, effective at communicating diverse emotions in aging.

### Age-related Difference in the Responses to Different Sensory Modalities and Specific Emotions

However, the main effect of age was tempered by a set of interactions, suggesting that age-related differences varied across modalities and across specific emotions. Specifically, in response to the visual and auditory stimuli, we found an age-related reduction in accuracy for negative expressions (i.e., fear, sadness, anger and disgust) and comparable performance for neutral and happy expressions. For the visual modality, this result is in accordance with individual studies using images of static faces showing different emotional expressions, which showed that certain discrete emotions, notably negative ones, are more sensitive to age-related variation (see for review, Ruffman et al., 2008). Studies regarding the auditory channel are more inconsistent because they are based on diverse paradigms. The results differ inasmuch as the studies did not isolate specific emotions (e.g., Orbelo et al., 2005; Mitchell, 2007; Mitchell et al., 2011), they investigated negative emotions only (e.g., Hunter et al., 2010), they explored a few contrasting emotions (e.g., Lambrecht et al., 2012) or they included several positive and negative emotions (e.g., Wong et al., 2005; Lima et al., 2014). Wong et al. (2005) found that older adults poorly recognized only sadness and happiness in speech; in contrast, using non-verbal vocalizations, Hunter et al. (2010) found that older adults poorly identified negative emotions (fear, anger, sadness, disgust), whereas Lima et al. (2014) found that older adults performed poorly for all emotions (positive and negative ones). Note however, that for scores, interpretations about age-related difference in responses to specific emotions are limited because of the presence of ceiling effects for happy and neutral expressions. Interestingly, for RTs the effects seem to be more general since older adults were especially slow to respond to all emotions.

These divergent results across aging studies may be due to the individual variability of the samples and the use of different types of emotional stimuli with varying presentation times, which might influence the identification of the given emotion. In the present study, the stimuli were presented or repeated until the subject provided a response. The observed slower RT for all negative emotions contrasts with the findings of recent studies (Pell and Kotz, 2011; Rigoulot et al., 2013) using verbal emotional stimuli, which showed that listeners are generally faster at identifying fear, anger, and sadness and slower at identifying happiness and disgust. This suggests that non-verbal affective vocalizations are processed at different rates. Interestingly, this time window is consistent with a work by Pell (2005); when happy, sad, or neutral pseudo-utterances spoken in English were cut from the onset of the sentence to last 300, 600, or 1000 ms in duration, emotional priming of a congruent static face was only observed when vocal cues were presented for 600 or 1000 ms, but not for only 300 ms. Hence, vocal information enduring at least 600 ms maybe necessary to presumably activate shared emotion knowledge responsible for multimodal integration. More importantly, our data show that the participants did not find it easier to identify emotion from isolated facial or non-verbal vocal cues. By contrast, Hunter et al. (2010), who used facial and vocal non-verbal emotions, found that emotion recognition was easier in response to facial cues than vocal cues. However, our experiment used not only negative but also happy and neutral expressions, which potentially improved the performance of older adults in both the visual and auditory modalities.

Overall, these results are consistent with the fact that age-related emotional difficulties do not reflect general cognitive aging (Orbelo et al., 2005) but rather a complex change affecting discrete emotions; notably, the same authors also suggest that the age-related decline in emotional processing is not explained by sex effects or age-related visual or hearing loss. Nevertheless, assessing hearing and seeing abilities objectively could have informed the pattern of our findings and we can consider the lack of measuring these covariates as a limitation of the study. For example, recent findings (Ruggles et al., 2011; Bharadwaj et al., 2015) suggest that despite normal or near-normal hearing thresholds, a significant portion of listeners exhibit deficits in everyday communication (i.e., in complex environments such as noisy restaurants or busy streets).

These results could also be interpreted in terms of the socio-emotional selectivity theory, which states that aging increases emotional control, diminishes the impact of negative emotions and facilitates concentration on more positive social interactions (e.g., Charles and Carstensen, 2010; Huxhold et al., 2013). However, Frank and Stennett (2001) have noted that using only a few basic emotion categories allows participants to choose their response based on discrimination and exclusion rules, which is less likely to be the case in a real-life setting. In particular, if happiness is the only positive emotion, participants can make the correct choice as soon as they recognize a smile. Therefore, a ceiling effect can be an alternative explanation to the socio-emotional selectivity theory. An alternative to examine possible valence-specific effects is the use of a similar number of positive and negative emotions (see Lima et al., 2014).

### Integration of Crossmodal Emotional Information in Aging Individuals

The principal goal of the current study was to explore whether older adults benefit from congruent crossmodal integration and to better understand the nature of this benefit. In daily life, the combination of information from facial and vocal expressions usually results in a more robust representation of the expressed emotion (e.g., De Gelder and Vroomen, 2000; Dolan et al., 2001; De Gelder and Bertelson, 2003), which thus results in a more unified perception of the person (Young and Bruce, 2011).

In our study, emotional faces and voices come from different sensory modalities to build a unified and coherent representation of the same percept (i.e., an emotion) as defined by crossmodal integration mechanisms (Driver and Spence, 2000). We showed that whereas older adults exhibited slower RTs under the crossmodal condition, resulting in a different temporal window of multisensory enhancement, a multisensory benefit occurred to the same extent in the two groups. However, early studies of multisensory integration in aging individuals showed that compared to younger adults, older adults did not benefit from multisensory cues (Stine et al., 1990; Walden et al., 1993; Sommers et al., 2005) and experienced a suppressed cortical multisensory integration response that was associated with poor cortical integration (Stephen et al., 2010). By contrast, more recent studies point toward an enhancement of multisensory integration effects in older adults, notably reporting shorter RT in response to multisensory events (e.g., Mahoney et al., 2011, 2012; DeLoss et al., 2013).

Consistent with the latter works, the present study indicates that in younger and older adults, emotional information derived from facial and vocal cues is not reducible to the simple sum of the unimodal inputs and suggests that multisensory integration is maintained with increasing age and could play a compensatory role in normal aging. This is in accordance with a magneto-encephalography study (Diaconescu et al., 2013), which indicated that sensory-specific regions showed increased activity after visual-auditory stimulation in young and old participants but that inferior parietal and medial prefrontal areas were preferentially activated in older subjects. Activation of the latter areas was related to faster detection of multisensory stimuli. The authors proposed that the posterior parietal and medial prefrontal activity sustains the integrated response in older adults. This hypothesis is supported by the theory of PASA and that of cortical dedifferentiation, stating that healthy aging is accompanied by decreased specificity of neurons in the prefrontal cortex (Park and Reuter-Lorenz, 2009; Freiherr et al., 2013). This could explain why the crossmodal RTs of older adults was longer than that of younger adults for each emotion.

Furthermore, a recent study using ERPs by Mishra and Gazzaley (2013) among healthy older adults (60–90 years old) suggested the existence of compensatory mechanisms susceptible to sustaining efficient crossmodal processing. The authors showed evidence that distributed audio-visual attention results in improved discrimination performance (faster RTs without any differences in accuracy in congruent stimuli settings) compared to focused visual attention. They noted that the benefits of distributed audio-visual attention in older adults matched those of younger adults. Interestingly, ERPs recoding during the task further revealed intact crossmodal integration in higher performing older adults, who had results similar to those of younger adults. As suggested by Barulli et al. (2013), attention, executive function and verbal IQ may play a role in the generation of a “cognitive reserve” that reduces the deleterious effects of aging and, thus, buffers against a diminished adaptive strategy (Hodzik and Lemaire, 2011). These results show the necessity of taking into account individual cognitive differences in aging. It is clear that significant cognitive decline is not an inevitable consequence of advancing age and that each cognitive domain is differentially affected. As aging can have diverse effects on cognitive functions, it is therefore important to emphasize the maintained functions rather than taking a customary approach that only underlines the loss of capacities among the elderly.

It should be noted however, as a possible limitation of the current study, that our stimuli are quite unnaturalistic since they combine non-dynamic (photographs) and dynamic (sound) stimuli. Although our participants did not report any incongruent perception of crossmodal stimuli, the use of emotional expressions that contain truly multimodal expressions (video and audio obtained from the same person), which are not posed, but enacted using the Stanislawski technique (see the Geneva Multimodal Emotion Portrayals, GEMEP; Bänziger et al., 2012) could be relevant.

## Conclusion

In conclusion, our results suggest that despite a decline in facial and vocal emotional processing with advancing age, older adults integrate facial and vocal cues to yield a unified perception of the person. Given the changes in facial and vocal modality exhibited by older adults, it may be helpful for family members and caregivers to use multiple sensory modalities to communicate important affective information. Thus, supplementing facial cues with vocal information may facilitate communication, preventing older individuals from withdrawing from the community and reducing the development of affective disturbances such as depression. Future research is required to further examine whether crossmodal integration can benefit older adults who exhibit cognitive impairments (e.g., Mild Cognitive Impairments, Alzheimer’s Disease). Such studies would be of particular interest in the context of recently developed assistive robotics platforms that prolong the ability of persons who have lost their autonomy to remain at home. For instance, serious games and socially aware assistive robots have actually been designed without considering the age-specific effects on social signal recognition. Therefore, improving the efficiency and suitability of these interactive systems clearly requires a better understanding of crossmodal integration.

## Author Contributions

Study concept and design was performed by LC, VL, MC, and MP. Data acquisition was conducted by VL. Data analysis was performed by LC, VL, and MC. All authors contributed to data interpretation and the final version of the manuscript, which all approved.

### Conflict of Interest Statement

The authors declare that the research was conducted in the absence of any commercial or financial relationships that could be construed as a potential conflict of interest.

## Footnotes

1 To control for potential gender differences, this variable was initially entered as a between-subject factor in the analyses. However, gender failed to yield any significant main effects (*F* < 1) or interactions (*p* > 0.1) so we collapsed across gender in the reported analysis.

2 Note that a ceiling effect was observed for happiness in both groups and for neutral in the younger group.

## Abbreviations

PASA: Posterior–Anterior Shift in Aging
fMRI: functional magnetic resonance imaging
ERP: event-related potentials
RT: response times
STS: superior temporal sulcus
p-STC: posterior superior temporal cortex
BDI: Beck Depression Inventory
MMSE: Mini Mental State Examination
CDFs: cumulative distribution functions.
